# Sustainable, Targeted, and Cost-Effective Laccase-Based Bioremediation Technologies for Antibiotic Residues in the Ecosystem: A Comprehensive Review

**DOI:** 10.3390/biom15081138

**Published:** 2025-08-07

**Authors:** Rinat Ezra, Gulamnabi Vanti, Segula Masaphy

**Affiliations:** 1 Chelmsford Campus, Anglia Ruskin University, Bishop Hall Lane, Chelmsford CM1 1SQ, UK; rinate6@gmail.com; 2 Rambam Health Care Campus (Medical Center), Haifa 3109601, Israel; 3 Multidisciplinary Research Unit, Karnataka Institute of Medical Sciences, Vidyanagar, Hubli 580021, India; nabisam1@gmail.com; 4 Faculty of Science and Technology, Tel Hai College, Upper Galilee, Kiryat Shmona 12210, Israel; 5 Applied Mycology and Microbiology, MIGAL-Galilee Research Institute, Kiryat Shmona 11016, Israel

**Keywords:** antibiotic residues, environmental sustainability, ecosystems, advanced laccase-based bioremediation, fungal laccases optimization, intermediates byproducts, toxicity evaluation

## Abstract

Widespread antibiotic residues are accumulating in the environment, potentially causing adverse effects for humans, animals, and the ecosystem, including an increase in antibiotic-resistant bacteria, resulting in worldwide concern. There are various commonly used physical, chemical, and biological treatments for the degradation of antibiotics. However, the elimination of toxic end products generated by physicochemical methods and the need for industrial applications pose significant challenges. Hence, environmentally sustainable, green, and readily available approaches for the transformation and degradation of these antibiotic compounds are being sought. Herein, we review the impact of sustainable fungal laccase-based bioremediation strategies. Fungal laccase enzyme is considered one of the most active enzymes for biotransformation and biodegradation of antibiotic residue in vitro. For industrial applications, the low laccase yields in natural and genetically modified hosts may constitute a bottleneck. Methods to screen for high-laccase-producing sources, optimizing cultivation conditions, and identifying key genes and metabolites involved in extracellular laccase activity are reviewed. These include advanced transcriptomics, proteomics, and metagenomics technologies, as well as diverse laccase-immobilization technologies with different inert carrier/support materials improving enzyme performance whilst shifting from experimental assays to in situ monitoring of residual toxicity. Still, more basic and applied research on laccase-mediated bioremediation of pharmaceuticals, especially antibiotics that are recalcitrant and prevalent, is needed.

## 1. Introduction

The use of antibiotic compounds to overcome bacterial infections has risen dramatically in the medical and agricultural sectors. However, non-utilized antibiotic compounds can enter the ecosystem and contaminate the environment. These then have a destructive effect on living organisms and the ecosystem. Thus, detoxification and removal of these antibiotic compounds from the environment using various physical, chemical, and biological treatments for the degradation of antibiotic compounds, including the elimination of the generated toxic end products, remain challenging. To address this, environmentally sustainable, green, and readily available bioremediation approaches for the transformation and degradation of these antibiotic compounds have been explored. Herein, we provide an overview of the following important issues, following this introduction (1): (2) the occurrence of antibiotic compounds in the environment; (3) antibiotic compounds’ effects on the ecosystem and non-target organisms, including the increase in resistance mechanisms; (4) biotransformation, degradation, and elimination of antibiotic residues in the ecosystem; (5) antibiotic bioremediation by fungal laccase; (6) enhancing laccase production and laccase optimization; (7) shifting towards real-world laccase-based bioremediation and ecotoxicity testing of its byproducts; (8) improving laccase-based bioremediation as well as environmental and economic sustainability; and (9) conclusions and future directions.

## 2. The Occurrence of Antibiotic Compounds in the Environment

The current state of knowledge concerning the use of antibiotics and their effects in both natural and human-controlled systems, including the latter to better understand the ecology and evolution of antibiotics’ role in combating microbial infection, has been widely reviewed. Indeed, the commercial application of natural and synthetic antibiotics to protect humans, animals, and plants from the microbial menace has increased steadily over the years [1,2]. Antibiotics of fungal or bacterial origin, as well as synthetic ones, are roughly divided into β-lactams, quinolones, glycopeptides, tetracyclines, aminoglycosides, macrolides, and sulfonamides (Table 1).

Antibiotics are widely used in human and veterinary medicine, aquaculture, agriculture, and other areas for therapeutic and non-therapeutic purposes [18]. Antibiotic compounds or their residues can enter the environment through various paths [19], such as hospitals, municipal, agricultural waste, sewage, surface water, and pharmaceutical plants [20]. It is estimated that 30–90% of the antibiotics administered to humans and animals are excreted in their native form into the environment, possibly due to their suboptimal metabolism in the body [21]. For example, β-lactam antibiotics in Asian aquatic environments have been reported at concentrations of nanograms per liter. Studies on β-lactam-resistant bacteria and resistance genes have been mostly conducted in China. The occurrence of these emerging contaminants is largely uncharted because many aquatic systems in the Asian region remain to be studied [22]. Table 1 lists the major antibiotic groups, their origin, applications, and concentrations found in the environment.

Moreover, some antibiotic residues, including quinolones, sulfonamides, and macrolides, have also been found in drinking water, increasing the human health risk [23,24]. These residues are pharmacologically and physiologically active and, even at very low concentrations, may adversely affect homeostasis in the human body [25].

## 3. Antibiotic Compounds’ Effects on the Ecosystem and Non-Target Organisms Including the Increase in Resistance Mechanisms

**Figure 1:**
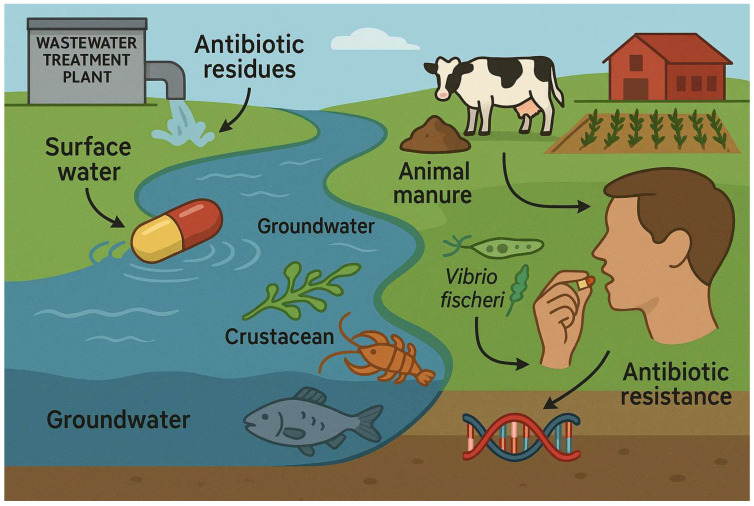
Schematic summary describing risks of residual antibiotic compounds on the ecosystem.

Antibiotics that are not completely metabolized by humans or animals can reach conventional wastewater-treatment plants (WWTPs), where they are difficult to completely remove. From there, they might contaminate the surface water and groundwater [26], thus leading to an increase in toxic effects on non-target organisms, food-chain contamination, a threat to bacterial environment sustainability, and some detrimental effects on the functioning ecosystem [27], and more, as shown in the schematic summary in Figure 1. Long-term exposure to these micro-residues could lead to irreparable damage to ecosystem niches and harmful mutations in living and non-target organisms [28,29]. For example, the most affected non-target aquatic species are the bacterium *Vibrio fischeri*, algae, crustaceans, and fish [30]. Moreover, antibiotic residues can adversely affect the homeostasis of soil microorganisms, including bacteria, viruses, and fungi, and inhibit their beneficial functions in organic matter turnover, soil-texture stabilization, and maintenance of soil fertility and plant root health [4,31,32].

An additional source of contamination is animal manure, which is used to fertilize vegetable crops and may contain commonly used veterinary medicines. Due to its high amounts in veterinary medicine and food additives, tetracycline is the most frequently detected antibiotic in animal manure [33]. Human consumption of these fertilized crops can lead to further toxic health effects, such as inflammatory reactions in the liver and detrimental effects on reproduction [30]. Antibiotic residues can also disrupt the production of short-chain fatty acids by gut-friendly bacteria in the human body [34].

Another concern is the increase in bacteria’s resistance to some antibiotics due to their long-term exposure to those antibiotics or other resistance mechanisms. Antimicrobial resistance (AMR) can be driven by antibiotic misuse or overuse in various sectors through various mechanisms, such as enzymatic modification and biofilm formation. Such mechanisms enable microbes to withstand the effects of antibiotics and their increased use [35]. With the increase in AMR, in recent years, there has been growing concern about the existence of antibiotic-resistance genes (ARGs). Emerging water contaminants are now being studied, including antibiotics, because of the global phenomenon of antibiotic resistance. The emergence of antibiotic resistance is a public health threat because, as bacteria become more resistant, infections increase. These infections require treatment with the more costly drugs as of last resort [22].

Notably, Barathe et al. (2024) [36] have indicated that such bacterial defense strategies against bactericidal agents can be transferred from antibiotic-resistant phenotypes to antibiotic-sensitive ones by well-characterized mechanisms, including horizontal gene transfer. Thus, ARGs and mobile genetic elements will help the bacteria survive under adverse conditions in all sectors, including freshwater systems.

Large numbers of ARGs have been detected in environmental samples, mainly in hospital sewage water and WWTPs, surface water, and soil [37,38]. In response to the increasingly widespread application of antibiotics, resistance can generally arise via two different mechanisms: (i) a de novo/first-time non-inherent mutation and (ii) ARGs in the environment may become less rare via the evolution of de novo/new resistance in response to anthropogenic selective pressures [34].

Ben et al. (2019) [39] recognized the emergence of ARGs as environmental pollutants [40]. The health threat to humans of antibiotic resistance associated with antibiotic residues in the environment consists mainly of the following: (i) the potential hazard of ingested antibiotic residues altering the human microbiome and promoting the emergence of and selection for resistant bacteria in the human body [41]; (ii) the potential hazard of creating selection pressure on the environmental microbiome. These potential hazards thus lead to reservoirs of ARGs and antibiotic-resistant bacteria, which are referred to as an environmental antibiotic resistance [42].

## 4. Biotransformation, Degradation, and Elimination of Antibiotic Residues in the Ecosystem

Biotransformation reactions, including the transformation of endogenous and exogenous molecules into active or inactive, more water-soluble chemicals, are fundamental. This involves some key biochemical drug-metabolizing processes, including oxidation, reduction, hydrolysis, and conjugation. Biotransformation of antibiotic residues can include destruction of their molecular structure through modification or hydrolysis using enzymes [43]. It can also involve changing the organic antibiotic residues from one form to another, which might reduce their toxicity and persistence [44]. For example, antibiotic compounds such as amoxicillin, ampicillin, and daptomycin have been bio-transformed to less toxic forms by eliminating the functional groups associated with their antibiotic activity using microbes in the wastewater for 20 h of incubation [45]. Such microbes are extremely adaptable and can even be found growing in contaminated regions, where they degrade or transform the antibiotic compounds [46].

Despite a reduction in the antibiotic compound concentrations in sewage-treatment plants or WWTPs, antibiotic residues may still be found in very small amounts in the wastewater bodies. Thus, it is a major challenge to remove them completely due to their stability in the face of conventional elimination methods. To overcome these challenges, several chemical, physical, and biological strategies have been examined for the removal of antibiotics and their residues [47]. Physical methods to remove these micropollutants include membrane-based processes and temperature degradation. Chemical methods include photocatalysis, ion exchange, ozonation, advanced chemical oxidation, filtration, cathodic degradation, plasma treatment, and coagulation [48]. In bioremediation, advanced oxidation processes (AOPs) have shown promising and efficient degradation of many types of organic pollutants and are the most studied degradation processes [49,50]. However, individual AOPs have some weaknesses, such as partial degradation of the organic moieties, high cost, the resultant secondary pollution due to the addition of catalysts and oxidants [51], and more. Hence, despite these methods’ promising results in removing pharmaceutical micro-residues, including antibiotic residues, there is a significant need to enhance their economic feasibility and efficacy in eliminating hazardous end products following antibiotic residue degradation.

Yan et al. (2019) [52] have compared different treatments with considerable removal efficiencies in respect to antibiotic residuals, including the following: 1. Advanced oxidation processes (AOPs), which attract attention due to their robust removal efficiencies but are cost-prohibitive for full mineralization of antibiotics and possibly produce sub-active toxic byproducts. 2. The adsorption processes and membrane technologies, which are satisfying but are unable to ultimately degrade antibiotics and are markedly impaired by the presence of other organic contaminants. 3. Biological methods for their highly versatile performance for in situ application but are usually time consuming.

Microbial and plant-derived methods are economically feasible and sustainable biological approaches that are in high demand for the efficient removal of micro-residues from the environment [53,54]. Microbial or plant-based bioremediation processes are usually conducted with whole organisms. However, the use of whole plants or microbes is usually time consuming, and they are sensitive to harsh wastewater or soil conditions, leading to loss of activity [55]. Hence, the use of purified enzymes is suggested.

## 5. Antibiotic Bioremediation by Fungal Laccase

### 5.1. Enzyme-Mediated Bioremediation with a Focus on Fungal Laccase

Efficiently removing micro-residues from the environment and the use of microbial enzyme-mediated bioremediation shows great potential. This is due to its catalytic action, which is environmentally friendly and economically feasible [56,57]. Enzyme-mediated bioremediation refers to the use of enzymes that occur naturally in microorganisms or plants to degrade or reduce harmful, undesirable, and recalcitrant environmental pollutants, thereby cleaning up contaminated sites. Enzymes are biocatalysts that lower the activation energy and facilitate the rapid and complete breakdown of substrates. Enzyme technology has been reported to enhance sensitivity and performance, thus providing a complete solution to problems that are associated with antimicrobial remediation [58,59].

The most common source of antibiotic-degrading enzymes is the fungal kingdom. The biotransformation of antibiotics by aerobic bacteria is rare, whereas fungi are better able to degrade or transform them aerobically. Of special interest are the white-rot fungi (WRF), which have been reported to degrade various antibiotic compounds (Table 2).

The WRF’s degradation activity is based on its ligninolytic enzyme composition and secretion as biocatalysts. Consequently, WRF and its ligninolytic enzymes have been widely applied in the removal of a range of contaminants, such as polycyclic aromatic hydrocarbons, pharmaceutically active compounds, endocrine-disrupting compounds, pesticides, synthetic dyes, and other environmental pollutants, including antibiotic compounds (Table 2) [57].

Different enzymes, but especially oxidoreductases (laccases), tyrosinases, and peroxidases, hold promise for the large-scale removal of antibiotic compounds from the environment [38]. Among the possible enzymes, laccase is widely used for the degradation of organic pollutants, including pesticides [66,67]. Laccases are widely distributed in plants, bacteria, and fungi, as exocellular enzymes that are secreted into the environment and play a role in lignin biotransformation [68,69]. In particular, WRF laccases have high potential to transform or mineralize a variety of organic pollutants and pharmaceutical products [70], serving as a biocatalyst or biosensor in the oxidoreductase reaction [71,72].

#### 5.1.1. Structural and Catalytic Characteristics of Laccases in Antibiotic-Based Bioremediation

**Figure 2:**
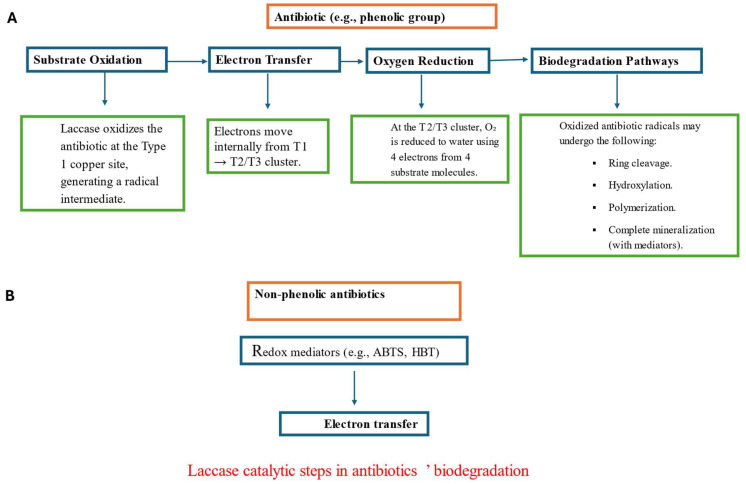
Schematic processes of laccase catalysis of antibiotic compounds biodegradation: (**A**) Without mediator. (**B**) With mediator.

Exploring how exocellular laccase plays a pivotal role in antibiotic biotransformation, it is necessary to understand the structural basis and the catalytic process that provides antibiotic bioremediation by laccases. Laccase is an oxidoreductase, catalyzing the oxidation and reduction reactions mainly to the various aromatic and non-aromatic compounds present in antibiotics [37]. As multicopper oxidases, laccases are typically monomeric glycoproteins of 60–80 kDa, composed of three cupredoxin-like domains (D1–D3) that contain four copper atoms arranged in three types: Type 1 (T1) copper that carries out the initial electron abstraction from the substrate while Type 2 (T2) and Type 3 (T3) coppers form a trinuclear cluster responsible for reduction of molecular oxygen to water [73]. The process has four steps: A. Substrate oxidation at T1, where phenolic or aromatic substrates lose one electron, forming a radical [74]. B. Electron transfer, where electrons pass from T1 to the trinuclear copper cluster (T2/T3). C. Oxygen reduction, where, after four electron transfers, O_2_ is fully reduced to 2H_2_O [73]. D. Substrate transformation, where the oxidized antibiotic undergoes ring cleavage, hydroxylation, or polymerization, depending on its structure [74]. Antibiotics that contain aromatic rings, amino groups, or phenolic moieties (like tetracycline) are oxidized directly by laccase-catalyzed oxidation [75,76], resulting in ring cleavage, hydroxylation, polymerization, and complete mineralization, whereas non-phenolic antibiotics (like fluoroquinolones) require redox mediators (e.g., ABTS, HBT) to facilitate electron transfer [77,78] (Figure 2).

The involvement of redox mediators, which are called the laccase-mediator system (LMS), can expand the substrate range of laccases to non-phenolic substances and improve their degradation efficiency.

#### 5.1.2. Laccase Antibiotic Degradation Efficiency

Bio-electrochemical systems (BESs), coupled with microbial metabolism and electrochemical redox reactions, have been considered promising alternatives for the degradation of antibiotic contaminants [52]. A comprehensive review of the effectiveness of antibiotic degradation by fungal laccases compared with new alternative methods, for example, Electro-Fenton (EF) or Bio-electro-Fenton (BEF) processes and Microbial Fuel Cells (MFCs), is presented in Table 3. Laccase could remove over 90% of certain antibiotics, such as fluoroquinolones, and over 80% sulfamethoxazole, using laccase-mediator systems [65,79]. While several new technologies show similar or higher efficiency of antibiotics degradation [80,81,82,83], the main advantage of laccase exploitation is that it is environmentally friendly, with low energy demand.

The promising role of laccases has been demonstrated in the food, paper, textile, and cosmetics industries, and its efficacious removal of environmental phenolic pollutants, such as antibiotics and pesticides, has also been shown [84,85]. Non-phenolic substrates can also be catalyzed by laccases through the involvement of redox mediators, which are basically low-molecular-weight organic compounds [86,87]. Thus, laccases have not only been shown to successfully remove pharmaceuticals alone [62,88] but also in combination with a mediator [89,90].

**Figure 3:**
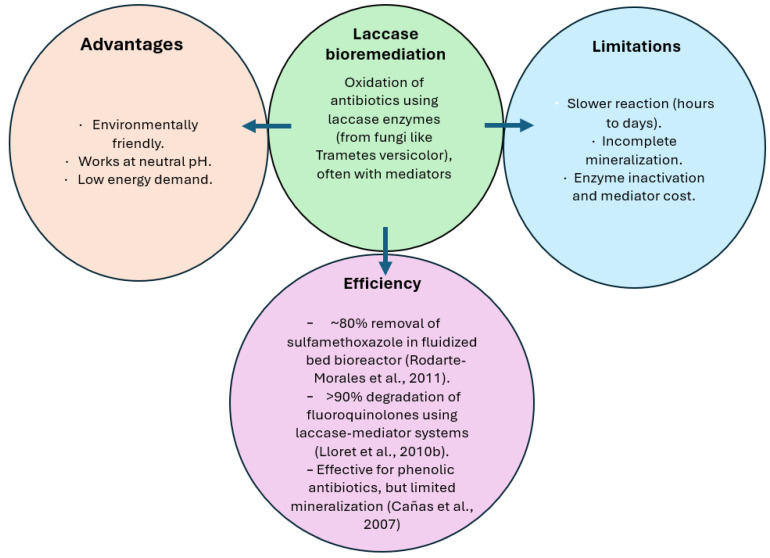
Laccase-based bioremediation of antibiotic compounds, including their efficiency, advantages, and limitations compared to other new emerging technologies summarized in Table 3 [75,76,79].

Altogether, the initial picture of laccase-based bioremediation, including its advantages, efficiencies, and limitations, is presented in Figure 3.

In the present review, more emphasis is given to the fungal laccase enzymes’ role in the degradation or biotransformation of pharmaceutical micropollutants in the environment rather than in the lab, including different methods, such as immobilization, gene expression, and regulation, which are used to improve their activity.

### 5.2. Laccase Immobilization Methods

Although laccase alone or in the presence of mediators may demonstrate good catalytic oxidation ability, free laccases are not stable. Enzyme immobilization is a common method to improve enzyme stability and confer reusability [91]. Accordingly, laccase immobilization has been reported in many studies [92,93].

Laccase immobilization technologies with different inert carrier/support materials have shown promise for the remediation of antibiotic residues in wastewater. The carrier materials that have been studied for enzyme immobilization thus far include both organic and inorganic carriers, based on their chemical composition. Organic carriers include cellulose, chitin, collagen, polystyrene, and polyacrylamides, among others, whereas inorganic carriers include silica, clay, kaolin, glass beads, metals, etc. [94]. In Olshansky et al.’s (2018) [94] study, laccase adsorbed to chitosan-modified clays showed higher activity than the enzyme alone. Cabana et al. (2011) [95] also showed that covalent binding of laccase to chitosan, using carbodiimide as the crosslinking agent, results in up to 260% higher laccase activity compared to free laccase. Higher stability for thermal and protease degradation was observed for laccase immobilized on mineral surfaces, and the protein remained active for longer periods than the free laccase [93].

Aricov et al. (2020) [96] presented a new laccase-immobilization protocol using wet chitosan microspheres and the non-toxic crosslinking agent 1-ethyl-3-(3-dimethylaminopropyl) carbodiimide hydrochloride (EDC). The proposed iterative protocol immobilized a large amount of active enzyme on the support. Compared to the free enzyme, the immobilized laccase was significantly more stable at various pHs and temperatures, had a longer storage life, and was better protected against thermal inactivation.

Different methods of immobilizing fungal laccases used for the degradation of antibiotics are listed in Table 4.

The most widely used method to immobilize laccase is adsorption. In particular, the use of a modified biochar to adsorb laccase has attracted much attention due to its excellent performance. In the study conducted by Wang et al. (2021) [91], laccase was immobilized by adsorption to cetyltrimethylammonium bromide–potassium hydroxide-modified biochar (CKMB), termed laccase@CKMB. Compared to free laccase, the storage and pH stability of laccase@CKMB were greatly improved. In addition, the laccase@CKMB could be reused for up to six reaction cycles while retaining 45.1% relative activity [91].

Laccase can also be physically adsorbed to a carrier through ionic bonding or covalent bonding. For example, laccase immobilized on graphene oxide or polyethersulfone can be reused for only one cycle [108], whereas laccase immobilized on a chitosan–clay composite is still effective after several uses [109]. Minor desorption and enhanced activity resulting from the laccase adsorbents suggested that a complex consisting of laccase and chitosan-modified S9 can be stable for several reaction cycles without losing substantial amounts of the enzyme to the reaction solution. The higher activity of adsorbed vs. free laccase was attributed to conformational changes in the protein’s structure [95].

Several studies have demonstrated the efficiency of immobilized laccase for antibiotic removal. In the study conducted by Garcia-Delgado et al. (2018) [103], laccase was immobilized on a tailor-made polyacrylonitrile–biochar composite nanofibrous membrane through covalent bonding. The obtained biocatalyst was used to remove chlortetracycline. The immobilized laccase was stable compared to free laccase and showed nearly 73% degradation of the antibiotic after 8 h of reaction [103]. This tailor-made membrane was also used for the removal of carbamazepine and diclofenac and showed 63.3% degradation of chlortetracycline [101]. According to Jeong and Choi (2020) [105], laccase immobilized on chitosan–tripolyphosphate beads showed the highest tetracycline-inactivation activity when ABTS was used as a mediator. Another approach was to prepare hollow mesoporous carbons tagged with laccase powder (L-HMC) obtained commercially from *Trametes versicolor.* L-HMS showed 90–100% efficiency in the degradation of tetracycline hydrochloride and ciprofloxacin hydrochloride from water bodies [110]. Similarly, Kadam et al. (2020) [111] fabricated halloysite nanotubes with chitosan and Fe_3_O_4_ nanoparticles coupled with *Trametes versicolor* laccase, and the composite, with mediators, succeeded in degrading sulfamethoxazole. Poly (methyl methacrylate) (PMMA) and magnetic nanoparticles were used to fabricate PMMA/Fe_3_O_4_ electrospun nanofibers. This material was used as a support to bind *Trametes versicolor* laccase for the degradation of tetracycline [112]. Immobilization techniques were also exploited when mediators were used. As such, laccase and ABTS were immobilized in an amino-functionalized metal–organic framework (MOF) composite membrane (PET/UiO-66(Zr)–NH_2_), resulting in a higher affinity for substrate and great recyclability [113].

## 6. Enhancing Laccase Production and Laccase Optimization

### 6.1. Screening for High-Laccase-Producing Sources and Optimizing Cultivation Conditions

Whilst more research is needed into larger-scale screening of high-laccase-producing sources, cultivation and production technologies to monitor the diversity and predict the efficacy of those enzymatic biocatalysts are enabling keys, and more active and potent enzymes should be used. Commercial application of laccase is often hampered by insufficient enzyme stocks, with very low yields obtained from natural sources. A range of methods to improve the acquisition of laccase activity was considered, including screening for high-laccase-producing sources and optimizing cultivation conditions [114]. For example, today’s omics technologies, including transcriptomics, proteomics, and metagenomics, can be utilized to develop methods for screening and researching the diversity and ecology of microorganisms and their possible uses in environmental bioremediation [115]. This also promotes the identification of key genes and metabolites involved in enhancing extracellular laccase activity [114].

The production of laccase activity is limited by various conventional approaches, such as heterologous expression, strain screening, and optimization of incubation conditions. There is an urgent need for a new strategy to meet industrial requirements more effectively and improve laccase activity. Attempts are still being made to develop novel approaches for further enhancing laccase activity. For example, Zhang et al. (2024) [114] have performed transcriptomic and metabolomic analyses to identify key genes and metabolites involved in extracellular laccase activity. Their findings indicated a strong correlation between the glutathione metabolism pathway and laccase activity. Subsequently, experimental verifications were conducted by manipulating the pathway using chemical approaches. The additive reduced glutathione (GSH) dose-dependently repressed laccase activity, while the GSH inhibitors (APR-246) and reactive oxygen species (ROS) inducer (H_2_O_2_) enhanced laccase activity. Their study suggests that laccase activity is negatively influenced by GSH metabolism and provides a theoretical basis for a novel strategy to enhance laccase activity by reprogramming glutathione metabolism at a specific cultivation stage.

### 6.2. Optimizing Laccase Production via Classical and Molecular Breeding Approaches

In addition to isolating natural, efficient laccase producers, classical and molecular breeding approaches are also used to increase laccase production:

A. Classical Breeding Approaches: Xu et al.’s (2016) [108] study aimed to improve laccase production by mutation of a *Coriolopsis gallica* strain and to determine the biological properties of the mutant. The high-yield laccase strain *C. gallica* TCK was treated with N-methyl-N-nitro-N-nitrosoguanidine and ultraviolet light. Among the mutants isolated, T906 was found to be a high-production strain of laccases. The mutant strain T906 showed three-times-higher laccase activity than the original strain TCK under optimized conditions. The extracellular laccase isoenzyme 1 was purified and characterized from the two strains, respectively, and their cDNA sequence was determined.

B. Molecular Breeding Approaches: Heterologous and Homologous Expression

B.1. Heterologous Expression:

Heterologous expression of laccase has been used to improve its acquisition and is invaluable in obtaining laccase proteins based only on metagenomic sequences [116,117,118]. Heterologous expression is also important for isolating a laccase from other isozymes, especially when the enzyme is not abundantly expressed or silent. In addition to structural and biochemical characterization, heterologously expressed laccases can be engineered by rational design or directed evolution for enhanced expression, catalytic activity, stability, etc. [118].

The earliest approach was rational design, which was used to modify the specificity of enzymes. More recently, directed evolution has proven to be a powerful tool for the modification of enzyme activities, which consists of the low-frequency introduction of randomly distributed mutations in a gene of interest, followed by selection of the mutated proteins possessing the desired properties. Efficient sampling of mutations, which is likely to affect enzyme function, has been conducted both experimentally and, on a much greater scale, computationally. This has resulted in remarkable improvements in substrate selectivity and specificity and the de novo design of enzyme activities within scaffolds of known structure [119].

Yang et al. (2017a) [118] have noted that enzyme resurrection, which is heterologous expression of ancestral enzymes reconstructed based on phylogenetic analysis and inference, is of particular interest [120]. Ancestral enzymes are likely to have unique and extreme properties, such as greater stability and substrate promiscuity than extant ones, considering characteristics of ancient life (e.g., thermophilic) and generalist–specialist conversion of enzymes during the course of evolution [121]. White-rot fungi and lignin degradation dated back to the Permo-Carboniferous period [122]; therefore, laccase resurrection brings an intriguing and promising toolset to laccase engineering and deserves more research efforts. The resurrected enzymes can then be subjected to further engineering by directed evolution. To ensure that these versatile enzymes meet industry standards and needs, they have been subjected to directed evolution and hybrid approaches that surpass the limits imposed by nature [120].

However, the laccase production achieved through heterologous expression is generally lower compared to that of native sources. For instance, the activity of hetero-expressed *Cerrena* sp. laccase was 20–50 times lower than that of native sources [123,124]. A similar phenomenon was also observed in the hetero-expression of *Trametes versicolor* laccase [125,126,127].

B.2. Homologous Expression

Due to low laccase yields in heterologous hosts, homologous gene expression in laccase-producing hosts might be of value for promoting laccase production. For overexpression, the laccase homologous gene is often driven by a strong promoter. The native laccase promoter was also used, and a high laccase production level of 1 g/L was achieved in the presence of 40 g/L ethanol after fermentation of transgenic *P. cinnabarinus* for 24 days [128].

In order to address the requirements of industrial-scale production of active and stable enzymes, and to increase their homologous gene expression, optimization of the culture medium components was also examined, e.g., adjusting the presence of metal ions, aromatic compounds derived from lignin, or the nitrogen [129].

Altogether, enhancing laccase yields is essential to lower production costs and promote industrial applications of the enzyme, but this also relies on an understanding of other elements leading to diverse expression patterns of laccase species, strains, and gene regulation.

### 6.3. Identifying Potential Elements Regulating Laccase Expression

Expression of laccase isozyme genes is differentially regulated throughout fermentation and in response to medium composition, such as metal ions, xenobiotics, as well as nutrient types and levels. Laccase expression analysis has been performed on mRNA and protein levels, and the distinct responses of laccase species, strains, and genes no doubt paint a complex picture of laccase expression regulation [118].

Following a successful screening of laccase-producing native hosts, laccase production is improved by fermentation technology development with respect to fermentation type, medium composition, and cultivation parameters [130]. Improved production can be achieved when focusing on the methods used for screening and by developing laccase activity assays and purification strategies.

Particularly, Elisashvili and Kachlishvili (2009) [131] have focused on several production approaches providing enhanced secretion of laccase enzyme by white-rot basidiomycetes (WRB), including the following: 1. Enzyme yield, which is species-dependent and strain-dependent, and selection of new organisms with tremendous synthesis of these enzymes. 2. Selection of the carbon source and lignocellulosic substrate, which play a crucial role in the enzyme production. 3. Targeting aromatic compounds that regulate the ligninolytic enzyme synthesis. Although their effect is very specific depending on the fungi’s physiological peculiarities, supplementing the medium at an appropriate concentration significantly accelerated *C. unicolor* laccase production and 4-fold increased the laccase specific activity. 4. Co-cultivation of appropriate fungi, which showed considerable promise as a strategy to highly enhance the enzyme production. For example, the pairing of *C. unicolor* and *Phellinus robustus* resulted in a 2-fold increase in the total laccase yield.

Still, the main issue delaying their implementation at an industrial scale is the low yield of ligninolytic enzymes in most white-rot fungi. Although many recombinant organisms efficiently overproduce various industrial enzymes, high expression of laccase and peroxidases in heterologous systems has not been achieved yet, and they still have to be obtained from natural sources [132,133].

### 6.4. Other Emerging Methods Enhancing Laccase Activity

Yet, there are new emerging methods of enhancing laccase activity for the potential industrial use of the enzyme. Choudhary et al. (2025) [134] have performed molecular docking studies on laccase (Pdb id: 1GYC) with 10 selected pharmaceutical compounds, including controls commonly present in wastewater. Their study aims to understand the binding interaction and stability of the enzyme–substrate complex to mediate the bioremediation of these pharmaceutical pollutants from wastewater. Docking analysis was performed using the Maestro Schrödinger suite. The result revealed a significant binding affinity of laccase with pharmaceutical pollutants ranging from 5 to 6 Kcal mol^−1^. Further, the 2-D analysis of ligands and polar amino acid residues unravels the involvement of hydrophobic interactions and the stability of enzyme–substrate complexes. The study suggested an effective laccase-mediated bioremediation method for wastewater treatment.

Another approach was challenged by Mora-Gamboa et al. (2024) [135], who had accurately predicted, using an in silico computational model, the behavior of laccase in degrading antibiotics whilst providing atomic-level insights into molecular interactions. Their present work aimed to “In Silico” simulate the use of *Ganoderma lucidum* GlLCC1 laccase in the degradation of antibiotics as contaminants. A 3D homology model of GILCC1, based on *Lentinus tigrinus* mushroom laccase, was used in order to model the utilization of *Ganoderma lucidum* GlLCC1 laccase in degrading antibiotics.

Furthermore, Mora-Gamboa et al. (2024) [135] have emphasized the importance of experimental assays and assessments of antibiotic degradation in wastewater, considering various chemical compounds. Martin et al. (2024) [136] have aimed to describe current and emerging methods for laccase activity assays and place them in the context of a potential industrial use of the enzyme. The already huge range of substrates of laccases can be further extended by the use of small molecules acting as mediators in the laccase-mediator system (LMS). To do so, however, it is essential to be able to quantify its activity into different matrices and on a maximum of substrates.

**Figure 4:**
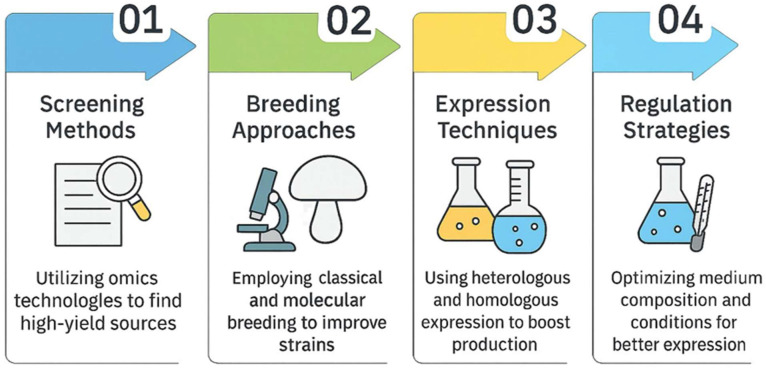
The challenges towards implementation at an industrial scale are presented.

In Figure 4, methods and challenges to enhance laccase production and challenges towards scaling up are summarized.

## 7. Shifting Towards Real-World Laccase-Based Bioremediation and Ecotoxicity Testing of Its Byproducts

### 7.1. Large-Scale and Cost-Effective Laccase-Based Applications

Several limitations to the use of laccase on an industrial scale still need to be overcome. Low productivity, low stability, limited reusability, and residual toxicity are the primary obstacles to the use of laccases in large-scale and cost-effective processes for industrial production and applications.

One of the major limitations for the large-scale application of laccase is the inability to produce large volumes of highly active enzymes at an affordable cost. The use of inexpensive sources for laccase production is being explored [137]. Although studies on optimization of growth conditions to enhance the production of microbial laccases for treating antibiotic contamination in wastewater were reported [138], commercially available purified laccase and large-scale composites are rarely marketed for the removal of pharmaceuticals.

Another concern is that previous studies have mainly focused on the removal of one or several compounds, usually at high concentrations, but this does not represent real-world environmental situations where multiple contaminants are present at low concentrations [62,88,99]. Those studies of antibiotic bioremediation in environmental settings were further constrained by the aforementioned limitations. Moreover, as most investigations were carried out in the laboratory, mostly using culture media in a controlled environment, there is no doubt that real-world environments present a more complicated structure and involve multiple factors that will impact the degradation process.

Challenging those limitations, several studies have shown the promising potential of using laccase in large-scale applications, including the removal of a broad range of antibiotics. Llorca et al. (2015) [139] and De Cazes et al. (2015) [140] demonstrated a promising technology to remove single compounds in laboratory-scale experiments using laccase immobilized on a ceramic support. Moving forward, they aimed to assess its performance in removing a broad range of 38 antibiotics on a reactor scale. In another study, a mixture of antibiotics at environmentally relevant concentrations was treated in an enzymatic membrane reactor with immobilized laccase with and without syringaldehyde as a mediator, allowing for affordable and large-scale applications [77].

In addition to the analysis of target chemicals to assess bioremediation efficiency, bioassays were used to investigate whether enzymatic treatment generates active transformation products that still have antibiotic activity or are toxic [141].

### 7.2. Laccase Bioremediation and Residual Toxicity

While studies reported that laccases could eliminate high ranges and concentrations of antibiotics, in a few cases, it is not always correlated with residual toxicity determination. For example, Becker et al. (2016) [141] have highlighted the residual toxicity in the presence of the mediator syringaldehyde (SYR) when immobilized laccase (*Trametes versicolor*) was used in removing a mixture of 38 antibiotics in an enzymatic membrane reactor (EMR) in a real-water reactor. Laccase + SYR degraded 32 of the antibiotics by >50% after 24 h. Residual toxicity, following degradation by laccase + SYR, was reported when the samples’ time-dependent increase in toxicity was tested in two bioassays, a growth inhibition assay and the Microtox assay [141]. Other investigators reported similar findings [142,143,144] by different bioassays. This is probably because the aromatic structures of syringaldehyde and antibiotics were oxidized into toxic byproducts, such as quinonoids or specific species, which could result in enhanced biotoxicity [145].

Challenging the detoxification of such toxic degradation products, in the study conducted by Zhang et al. (2020a) [146], bacteriostatic assays and survival testing of *Escherichia coli* and *Bacillus subtilis* (*B. subtilis*) confirmed the loss of antibiotic activity for tetracycline and ampicillin, as well as the low ecotoxicity of the degradation products [146]. Herein, to overcome such toxic degradation, Zhang et al. (2020a) [146] utilized effective in situ biomineralization methods to immobilize *B. subtilis*-derived laccase into the copper-Trimesic acid framework (Cu-BTC) and where the synthesized Lac-case@Cu-BTC particles were used to degrade tetracycline and ampicillin.

**Figure 5:**
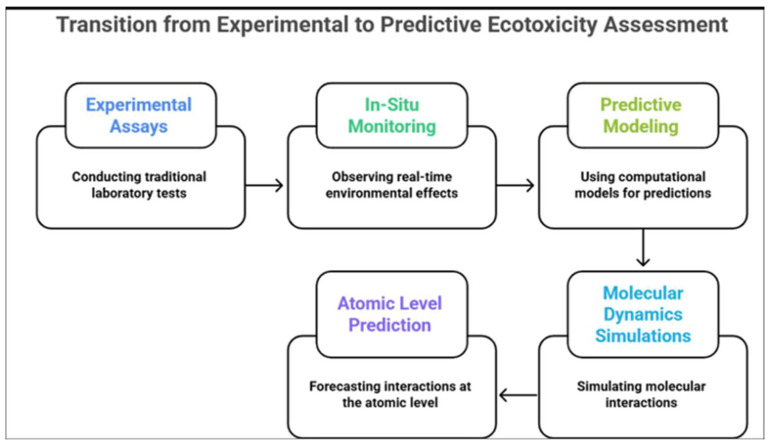
Shifting from experimental assays to in situ monitoring in ecotoxicity assessment.

Experimental assays or tests under controlled conditions, including Microtox and bacterial assays [141], bacteriostatic assays and survival testing [146], and growth inhibition assays or antimicrobial activity tests [86,103] are universally used. Yet, emerging in situ methods can provide more accurate insight into laccase final or intermediate biodegradation products and interactions for real-world simulation and larger models (Figure 5). From a predictive level, it is suggested to apply ‘Molecular dynamics simulations’ using 3D ‘in silico’ computational models, accurately predicting or simulating, on an atomic level, the molecular interactions of antibiotic degradation in wastewater, considering various chemical intermediates or final compounds, [135].

### 7.3. Exploring Alternative Eco-Friendly and Cost-Effective Enzymatic Systems

Whereas the toxicity of laccase in combination with the mediator syringaldehyde (SYR) has been noted above, laccases can oxidize an exceptional range of natural substrates (e.g., polyphenols, benzenethiols, aromatic or aliphatic amines, and many others). Natural substrates and mediators where the toxicity of their intermediate or final breakdown products of antibiotics after laccase treatment should be addressed. Although their mechanisms appear attractive, many of the synthetic mediators used so far are maligned by high cost, possible inhibitory effects on enzyme activity, and the creation of seemingly toxic metabolites, thereby motivating the search for natural forms [147,148].

Different natural compounds that might act as alternative mediators were reported. Laccases from unrelated organisms react differently per mediators and/or substrates [149]. Although approximately 100 unique potential mediator compounds have been enlisted for the LMS, ABTS and HBT remain the most patronized [149,150]. Some natural mediators include aniline, 4-hydroxybenzoic acid, and 4-hydroxybenzyl alcohol [150,151] alongside 3-hydroxyanthranilic acid (HAA), detected in the broth of *P. cinnabarinus* [152,153]. Interestingly, some of these unconventional mediators have demonstrated almost equal reliability as the commonly used ABTS and HBT [150]. Phenol red and derivatives, especially dichlorophenol red, are mediators of *Poliporus pinsitus* laccase, whose oxidation quotient of the nonphenolic substrate 4-methoxybenzyl alcohol was reported to be at least 10 times higher than with HAA [151].

Morozova et al. (2007b) [154] hinted that most compounds are not worthy “laccase mediators” as ascribed, due to their electrochemically unsteady oxidized intermediates, thereby eliciting a meager amount of redox cycles per catalytic oxidation of non-phenolic compounds. There has been no aggressive employment of LMS at a large scale due to the earlier stated reasons. However, the adoption of naturally occurring laccase mediators would present the “light at the end of the tunnel” for in situ environmental applications [148].

Challenging the high costs associated with enzymatic oxidation that are due to the cost incurred on materials, namely, laccase enzyme and mediators like ABTS, Mathur et al. (2021) [138] demonstrated that the total cost of treatment could be reduced by 71.7%. This is demonstrated when commercial-grade laccase was replaced by crude enzyme extracted from indigenous macrofungi isolates such *Pleurotus eryngii*, *Pleurotus florida*, and *Pleurotus sajor caju* [138]. Their results indicated that the crude laccase system from the indigenous fungal isolates has the ability to degrade more than 88% of levofloxacin at 5 μg mL^−1^ in both waste and pharmaceutical wastewater, which is a far higher antibiotic concentration than what is detected in real environmental matrices. Still, making it effective when dealing with real wastewater contaminated with antibiotics, future studies on optimizing the crude enzyme quantity to achieve near 100% degradation of levofloxacin are being carried out by Mathur et al. (2021) [138], to make the treatment commercially viable as an alternative to conventional treatments.

## 8. Improving Laccase-Based Bioremediation and Environmental and Economic Sustainability

The high costs of laccase and mediators remain a key shortcoming for the enzymatic treatment of antibiotic-contaminated wastewater at a larger scale [113]. Another urgent topic is the improvement of immobilized laccase’s stability and longevity using novel techniques and other promising support materials. Sustainability, such as the use of non-ecotoxic and biodegradable materials, should be considered in the future. More research should be performed on the underlying mechanisms of laccase-catalyzed pollutant degradation, focusing on the complex interactions among laccase, the supporting polymers, and the pollutants [155]. Herein, Sun et al. (2024) [142] have examined in detail the mechanisms for immobilized laccase-evoked removal of antibiotics, owing to immobilization technology reinforcing the stability and reusability of free laccase in bioremediation. In particular, advances in laccase-provoked transformation mechanisms (i.e., oxidative decomposition and radical polymerization) of antibiotics are highlighted, and a valuable guideline beneficial to large-scale practical applications is provided by [142].

It should be noted that the smaller size of enzymes relative to microbial cells facilitates their direct contact with the contaminants, leading to rapid and effective degradation or reduction to an admissible or less harmful state. In addition, advances in nanotechnology coupled with enzyme technology have introduced a new concept of single-enzyme nanoparticles to treat pollutants or contaminants. These are more productive, selective, and faster than enzyme treatment alone [58].

Besides improving the technologies for enzyme-based applications, future research might focus on lowering the cost of laccase production by searching for effective methods to isolate the enzymes from the fungi on a larger scale. However, before moving to commercial-scale fungal enzyme isolation, purification, and production, a search for new laccases with higher biocatalytic activity and stronger affinity, enabled by the supporting materials, is called for. The search for more effective laccases could be conducted using metagenomic data along with cloning and gene-editing technologies [155]. Specifically, various in silico methods, utilizing bioinformatics tools, have been employed to identify such enzymes [156]. Recently, the cloning of laccase has been reported from sequences obtained from the metagenomic data of tannery wastewater [157]. The laccase sequence was transformed into *E. coli* Rosetta (DE3) and harvested to produce thermohalotolerant laccase [157]. Various genetic mutations have been employed as a strategy to yield more effective laccase from simple random mutations using UV light exposure (*Pleurotus ostreatus*) [158] to a more complex protocol of error-prone polymerase chain reaction (PCR) and site-directed mutagenesis (*Bacillus licheniformis*) [159].

While laccases have been broadly applied as multitasking biocatalysts in various industries, their activity tends to be limited by easy deactivation, lack of adequate stability, and susceptibility under complex conditions. Identifying stable laccase as a green biocatalyst is crucial for developing cost-effective biorefining processes. In this vein, Ariaeenejad et al. (2022) [160] attempted in silico screening for a stable metagenome-derived laccase (PersiLac1) from tannery wastewater in a complex environment. Their laccase exhibited high thermostability, retaining 53.19% activity after 180 min at 70 °C, and it was stable in a wide pH range (4.0–9.0). After 33 days of storage at 50 °C, pH 6.0, the enzyme retained 71.65% of its activity [160].

Recently, biogenic nanoparticles fabricated using microorganisms have gained much importance. Their unique properties like high surface area and high catalytic reactivity make them a potent candidate for biodegradation and biosorption of emerging contaminants. Owing to their biological nature and nontoxicity, they have emerged as a sustainable alternative to chemically synthesized nanoparticles. Apart from this, enzymes immobilized on nanomaterials have also received considerable research attention. Immobilization is a promising approach to improve enzyme activity, stability, and reusability [161].

## 9. Conclusions and Future Directions

Laccases, particularly fungal laccases, can provide a sustainable solution for the degradation of diverse hazardous pollutants/contaminants into less toxic or non-toxic compounds. The contaminants investigated to date in the context of their removal by laccase from water bodies are not limited to antibiotics and include anti-analgesics, anti-depressants, biocides (e.g., atrazine, pentachlorophenol, isoproturon, metolachlor, spinosad, triclosan), endocrine-disrupting agents (bisphenol A), steroid hormones, and microplastics. Nevertheless, there are several challenges to overcome for a large-scale application to reduce the antibiotics in wastewater. Despite the advantages, enzymatic remediation also has certain limitations that restrict its application to bioremediation processes, such as the production cost of the enzymes and their stability and activity interacting with a wide range of contaminants. Since the beginning of the 21st century, specific features of bacterial and fungal laccases have been exhaustively adapted in order to meet the industrial demands for high catalytic activity and stability in conjunction with reduced production cost. Among the goals established for laccase engineering are heterologous functional expression, improved activity and thermostability, tolerance to non-natural media (organic solvents, ionic liquids, physiological fluids), and resistance to different types of inhibitors. These are all challenges that have been met when obtaining a more comprehensive understanding of laccase structure–function relationships [129].

Comprehensive research is still needed in the following: (i) the use of laccase-based products alone or in combination with mediators that could further enhance the degradation of micropollutants; (ii) the optimization of laccase-based applications, improving their stability, reusability, and efficacy, to be shifted toward larger-scale purification and production of a wide range of sensitive enzyme-based applications; and (iii) the toxicity of intermediate or final breakdown products of antibiotics after laccase bioremediation, whilst shifting from experimental assays to in situ monitoring of residual toxicity.

Altogether, basic and applied laccase research with an emphasis on laccase-mediated bioremediation of pharmaceuticals, especially antibiotics from a broad class of emerging organic contaminants that are recalcitrant and prevalent, is needed. With low laccase yields in natural and genetically modified hosts continuing to constitute a bottleneck for industrial-scale applications [118], in situ methods for enzymatic bioremediation of antibiotic residues in the environment remain a challenge.

## Figures and Tables

**Table 1:** Antibiotic groups, their origin, applications, and concentrations in the environment.

Antibiotics	Origins	Applications	Concentration in the Environment	References
ꞵ-Lactams	Fungi and bacteria	Humans, veterinary	1.67 µg/L (effluent)	[3,4]
Tetracyclines	Bacteria	Humans, veterinary, agriculture	6.8 mg/L	[5,6]
Quinolones	Synthetic	Humans, veterinary, agriculture, aquaculture	3.35 µg/L (effluent)	[4,7,8]
Sulfonamides	Synthetic	Humans, veterinary	0.326 µg/L (river water)	[9,10,11]
Macrolides	Bacteria	Humans, veterinary	3.847 µg/L (wastewater treatment plants)	[12,13,14]
Amino-Glycosides	Bacteria	Humans, veterinary	3.4 µg/L	[15]
Glycopeptides	Bacteria	Humans	0.0127 µg/L	[16,17]

**Table 2:** White-rot fungi used to degrade antibiotics in vitro.

Antibiotic Class	Antibiotic Group	White-Rot Fungi Used to Degrade Antibiotics	Reference
Fluoroquinolone	Ciprofloxacin	*Pleurotus ostreatus*	[60]
Fluoroquinolone	Levofloxacin	*Coriolopsis gallica*	[61]
Quinolones	Norfloxacin	*Trametes versicolor*	[62]
Tetracycline	Oxytetracycline	*Trametes versicolor*	[63]
β-Lactams	Ampicillin	*Verticillium leptobactrum*	[64]
Sulfonamides	Sulfamethoxazole	*Phanerochaete chrysosporium*	[65]
Aminoglycoside	Neomycin	*Trametes versicolor*	[61]

**Table 3:** Laccase-mediated antibiotics bioremediation compared with other new alternative methods in terms of mode of action/mechanism, efficiency, advantages, and limitations.

Characteristics	Bioremediation Methods:			
	Electro-Fenton (EF)	Bioelectro-Fenton (BEF)	Microbial Fuel Cells (MFCs)	Fungal Laccase Bioremediation
Mode of action/ mechanism:	EF generates hydroxyl radicals (•OH) from electro-generated H_2_O_2_ and Fe^2+^ in acidic conditions. These radicals degrade antibiotics non-selectively.	Similar to EF, but H_2_O_2_ is produced biologically by microbes at the cathode, reducing external chemical input.	Electroactive bacteria degrade antibiotics while generating electricity. Oxygen or alternative acceptors reduce compounds at the cathode.	Oxidation of antibiotics using laccase enzymes (from fungi like Trametes versicolor), often with mediators.
Efficiency:	Removal rates >90% for many antibiotics (ciprofloxacin, sulfamethoxazole, amoxicillin) in <1–2 h. · High mineralization >70% total organic carbon (TOC) removal. Hospital wastewater treated via EF achieved 93% removal of antibiotics and a substantial reduction in resistance genes [80].	Achieved 85–95% removal of tetracycline and oxytetracycline using BEF in <3 h [81]. · Less energy-intensive than EF. · Capable of treating low-concentration antibiotics in real wastewater.	MFCs removed ~70–80% of sulfamethoxazole and tetracycline over several days [82]. · Removal efficiencies vary but can reach 90% with optimized biofilms and operating conditions [83].	~80% removal of sulfamethoxazole in fluidized bed bioreactor [75]. >90% degradation of fluoroquinolones using laccase-mediator systems [79]. Effective for phenolic antibiotics, but limited mineralization [76].
Advantages:		· Lower energy and chemical cost. · Integrates biological and electrochemical processes. · Better suited for longer treatment times and eco-friendly applications.	· Simultaneous energy production. · Sustainable and low-energy. · Less chemical input compared to EF.	·Environmentally friendly. · Works at neutral pH. · Low energy demand.
Limitations:			· Slower than EF/BEF. · Lower mineralization. · Sensitive to environmental fluctuations.	· Slower reaction (hours to days). · Incomplete mineralization. · Enzyme inactivation and mediator cost.

**Table 4:** Different fungal laccases used for the degradation of antibiotics and its immobilization method.

Fungi	Enzyme	Matrix	Method	Compound	References
*Pleurotus eryngii*	Laccase	Microcapsule	Immobilization (encapsulation)	Tetracycline	[97,98]
*Aspergillus oryzae*	Laccase	Granular activated carbon	Immobilization (adsorption)	Sulfamethoxazole	[99]
*Trametes versicolor*	Laccase	Magnetic silica microbeads	Immobilization (covalent binding)	Acetaminophen (Paracetamol)	[100]
*Trametes versicolor*	Laccase	Polyacrylonitrile-biochar composite nanofibrous membrane	Immobilization (adsorption)	Chlortetracycline	[101]
*Cerrena unicolor*	Laccase	Magnetic nanoparticles cross-linked to laccase	Immobilization (covalent binding)	Tetracycline, Oxytetracycline, Ampicillin, Sulfamethoxazole Erythromycin	[86]
*Phanerochaete chrysosporium*	Laccase	-	Liquid-phase batch experiments	Tetracycline and sulfathiazole	[102]
*Trametes versicolor*	Laccase	Biochar and stevensite	Immobilization (covalent binding)	Tetracycline	[103]
*Trametes versicolor*	Laccase	Bentonite-derived mesoporous material	Immobilization (adsorption)	Tetracycline	[104]
*Trametes versicolor*	Laccase	Chitosan tripolyphosphate beads	Immobilization (covalent binding)	Tetracycline	[105]
*Pycnoporus* sp.	Laccase	-	Batch culture	Tetracycline	[106]
*Trametes hirsuta*	Laccase	-	Batch culture	Chloramphenicol	[107]
*Coriolopsis gallica*	Laccase	-	Solid and liquid media	Levofloxacin	[61]

## Data Availability

No new data were generated or analyzed in support of this research.
